# Wearable Biomonitoring Platform for the Assessment of Stress and its Impact on Cognitive Performance of Firefighters: An Experimental Study

**DOI:** 10.2174/1745017901814010250

**Published:** 2018-10-31

**Authors:** Susana Rodrigues, Joana S. Paiva, Duarte Dias, Gonçalo Pimentel, Mariana Kaiseler, João Paulo S. Cunha

**Affiliations:** 1 Institute for Systems Engineering and Computers – Technology and Science (INESC TEC), Porto, Portugal; 2 Faculty of Engineering (FEUP), University of Porto, Porto, Portugal; 3 Astronomy and Physics Department, Sciences Faculty, University of Porto, Porto, Portugal; 4 Institute for Sport, Physical Activity and Leisure, Leeds Beckett University, Leeds, UK

**Keywords:** Biomonitoring platform, Stress, Heart rate variability, Firefighters, Cognitive performance, Firefighter

## Abstract

**Background:**

Stress is a complex process with an impact on health and performance. The use of wearable sensor-based monitoring systems offers interesting opportunities for advanced health care solutions for stress analysis. Considering the stressful nature of firefighting and its importance for the community’s safety, this study was conducted for firefighters.

**Objectives:**

A biomonitoring platform was designed, integrating different biomedical systems to enable the acquisition of real time Electrocardiogram (ECG), computation of linear Heart Rate Variability (HRV) features and collection of perceived stress levels. This platform was tested using an experimental protocol, designed to understand the effect of stress on firefighter’s cognitive performance, and whether this effect is related to the autonomic response to stress.

**Method:**

The Trier Social Stress Test (TSST) was used as a testing platform along with a 2-Choice Reaction Time Task. Linear HRV features from the participants were acquired using an wearable ECG. Self-reports were used to assess perceived stress levels.

**Results:**

The TSST produced significant changes in some HRV parameters (AVNN, SDNN and LF/HF) and subjective measures of stress, which recovered after the stress task. Although these short-term changes in HRV showed a tendency to normalize, an impairment on cognitive performance was found after performing the stress event.

**Conclusion:**

Current findings suggested that stress compromised cognitive performance and caused a measurable change in autonomic balance. Our wearable biomonitoring platform proved to be a useful tool for stress assessment and quantification. Future studies will implement this biomonitoring platform for the analysis of stress in ecological settings.

## 1 INTRODUCTION

 Stress is widely recognized as a global challenge and has been the focus of concern by many researchers. Stress results from a transaction between the individual and the environment, where the individual perceives the demands of a situation as exceeding their individual resources [1]. Stress perceptions are likely to activate physiological responses. Cannon was one of the first physiologists to explore the impact of stress, by describing the instinctual “fight-or-flight” response [2]. When a threat is perceived, the Autonomic Nervous System (ANS) is triggered: the parasympathetic nervous system activation is reduced and the sympathetic nervous system is overactivated. This results in the secretion of stress-related hormones which leads to potential vasoconstriction of blood vessels, increased blood pressure, increased muscle tension, changes in Heart Rate (HR) and a decrease in heart rate variability (HRV). More recently, other possible response to threat exposure, called “Freezing”, has been investigated [3]. This response has been more evident in non-human species. It is characterized by bodily immobility and reduction in HR [4]. In sum, when facing a threat, both the sympathetic and parasympathetic branches of the Autonomic Nervous System (ANS) are activated. While freezing is related with dominance in parasympathetic activity, fight-or-flight reactions are characterized by parasympathetic withdrawal and sympathetic elevation [5].

The impact of the stress in the health condition is currently well recognized. Several personal, organizational, and medical costs, are being associated with increased stress health problems, such as heart disease, hypertension, upper respiratory tract infections, peptic ulcers, reduced immunity, migraines, alcoholism, depression, suicidal tendencies, anxiety, as well as other mental disorders [6, 7]. For these reasons, it is extremely important to have instrumental solutions that allow the stressful event assessment and monitoring in order to measure, quantify and correlate with health impact. The use of technological wearable devices such as biomonitoring mobile sensors, that allow the continuous monitoring of physiological signals, offer interesting opportunities for advanced personal health care solutions and analysis of stress [8, 9]. HRV has been proposed as a feasible and reliable quantitative marker describing the activity of the ANS in relation to stress [10]. The HRV defines the complex variation of beat-to-beat intervals mainly controlled by ANS through the interaction of sympathetic and parasympathetic activity [11]. However, little agreement exists in identifying the best HRV classifiers for stress [12].

The current study sought to investigate stress impact on a sample of firefighters, since firefighting is a very stressful and demanding occupation [13, 14]. As an example, during emergency scenarios, firefighters have to quickly respond to innumerous stimuli under pressure and the decisions made during these emergency situations tend to be based on information that is ambiguous, incomplete or unusual, further complicating the decision process [15]. Hence, stress is not only associated to physiological reactions, but also to cognitive responses such as attentional processes [16]. Empirical evidence investigating the relationship between stress and cognitive performance has been quite mixed, depending on the levels of stress, nature of task, individual variables and so forth [17]. There are studies proposing that stress can negatively affect cognitive performance, particularly in searching for alternative solutions and making correct decisions [18]. As opposed, other studies suggested that stress enhances the ability to better respond to stressful situations [19]. Pioneering work in this area was developed by Yerkes and Dodson [20]. These authors proposed the Yerkes-Dodson Inverted-*U* function relating arousal and performance. Accordingly, performance increases with physiological or mental stress, but only up to a certain point. When the level of arousal is too high, performance decreases [21]. At the optimal level of arousal with regard to performance, stress is supposed to be minimal since adaptation to performance demands is maximal.

Considering that it is difficult to evaluate stress during firefighter’s real-life emergency, experimental designs are one typical alternative method. Advantages of such designs include avoiding retrospective report problems and can add rigor of an experimental design as well as allowing physiological stress response monitoring [22].

Following the above state of the art technology and considering the importance of investigating the influence of stress on health and cognitive performance, a controlled experimental protocol, using a wearable biomonitoring platform was designed for this effect. Hence, two hyphotheses were formulated: **Hypothesis 1.** Acute stress would cause a significant reduction in some Electrocardiogram (ECG) time-domain parameters—AVNN; SDNN; RMSSD; pNN20; pNN50 and an increase in ECG frequency-domain—LF/HF. Our second hypothesis was as follows: **Hypothesis 2.** Acute stress would impair cognitive performance, by increasing the Reaction Times (RT) and percentage of missing answers.

## 2 METHODS 

### 2.1 Participants

Twenty-one firefighters (2 females and 19 males) from a Fire department in Portugal voluntarily participated in this study. The age range was 21 to 59 years (M =29.90 ± 8.83). Regarding the educational background, seven had completed primary school, 10 completed the secondary school and four had attained graduation level education. The years of practice ranged between less than one year to 29 years (M=8.33 ± 8.21). The exclusion criteria for the study were participants having a history of cardiovascular disease and/or taking prescription drugs known to affect cardiovascular function. The study was approved by the University of Porto Ethics Committee. All informants were carefully instructed about the study protocol and gave written informed consent prior to examination.

### 2.2 Experimental Setup

For the acquisition of physiological data, participants were equipped with Vital Jacket® [23, 24]. The Vital Jacket® is a wearable bio-monitoring platform (in form of a t-shirt) able to collect medical grade ECG signals in real-time, without affecting daily activities of users. It also contains a 3-axis Accelerometer system, allowing ECG signals correction for actigraphy profiles and a Bluetooth transmitter that enables the visualization of ECG signal in real-time and also saves all the data in a SD memory card.

This equipment is certified according to the MDD93/42/EEC medical device directive and holds the European Conformity medical device mark [25]. An android smartphone application was used for data synchronization and event marking. This system pairs with the Vital Jacket^®^ *via* Bluetooth and enables the exact annotation of events in the device, using “Radiobuttons”. These events are saved in the device and synchronized with the ECG that is being acquired in real time. The android application stores all the information about the events in an SQL Light DataBase, from where a report of the event data can be generated and exported for further air traffiprocessing and analysis, always ensuring the synchronization between those events information and the data collected by the Vital Jacket®. Fig. (**1**) demonstrates the bio monitoring platform architechtutre.

In order to gather psychological stress data, the Spielberg State-Trait Anxiety Inventory (STAI 6-item short-form) was used [26]. This instrument allows to quantify emotional, physical, and cognitive aspects of stress. Additionally, Visual Analogue Scales (VAS) were used to assess perceived stress [27]. Participants have to rate the average level of perceived stress experienced during the tasks by marking any point on a 10-centimeter line ranging from ‘None’ to ‘As bad as it could be’.

Demographic and medical surveys were also used in order to assess participant’s current health state. Cognitive performance data was calculated using Matlab® scripts developed by our team.

### 2.3 Procedure

A presentation session was organized to explain the aim and the overall protocol of the study. Then, those who wanted to participate in the study signed their name in a paper, expressing therefore their will to participate. The protocol lasted approximately 50 minutes and included a combination of tasks as shown in Fig. (**2**).

Participants completed the demographic, medical survey and STAI 6-item short-form. Then, they were equipped with Vital Jacket® and were allowed to sit comfortably for a 5-minute resting baseline. Additionally, at the beginning of the protocol and at the end of each task, firefighters were requested to fill in the VAS in order to assess perceived stress. Then, they started by performing a 2-Choice Reaction Time Task (CRTT) [28]. This task required participants to respond to a stimulus, by clicking in a button, as fast as possible. The stimuli consisted in three different types of targets: (a) an arrowhead pointing to the left on top of a square both filled with a sinewave grating pattern; (b) the same stimulus, but with the arrowhead directed to the right; (c) the grating square only (without an arrowhead) which remained in the center of the screen, between the appearance of each stimulus along the task. The stimuli were generated with Matlab®. The stimuli selected were subtle enough to induce incorrect or missed responses by the subjects during attention lapses caused by fatigue. This task was performed twice, at the beginning of the protocol (CRTT 1; pre-stress condition) and after the stress task (CRTT 2; post-stress condition). The stress procedure was based on an acute psychosocial stress paradigm, the Trier Social Stress Test (TSST) [29]. The TSST inlcude elements of public speaking, mental arithmetic and anticipation. The period of induced stress lasts approximately 15 minutes, and is divided into 5-minute components. Before the test begins, the participants are fitted with the wearable ECG equipment. Stress induction begins with the participant being taken into a room where a panel of an evaluation comitee that includes three judges, along with a video camera and audio recorder. The first 5 minute component is the anticipatory stress phase, during which the judges ask the participant to prepare a 5 minute presentation. This participation is framed as part of a job interview. Also, the judges have been trained to maintain neutral expressions throughout the test. The participant is permitted to use paper and pen to prepare their presentation, but this paper is then unexpectedly taken away from them when it is time to begin the presentation. During the 5 minute presentation component, the judges observe the participant without comments. If the participant does not use the entire 5 minutes, they will ask him/her to carry on, until the entire 5 minutes have been used. The presentation is immediately followed by the mental arithmetic component, during which the participant is asked to count down from 1022 by 13´s for 5 minutes. When making a mistake, the participant is asked to start over. Immediately after the test, there is a debriefing, in which the participant is told that the purpose of the test was to evoke stress, and that the results are in no way a reflection on his or her personal abilities. This task was selected for inducing stress, considering its components of unpredictability and uncontrollability, as a means to represent those elements of stressors that are encountered daily by firefighters.

During all session, one of the researchers used the android smartphone application to mark the events that synchronized with the ECG. At the end of the protocol participants filled in STAI 6-item short-form again.

### 2.4 Data Analysis

#### 2.4.1 Statistics

Data were statistical analyzed using IBM SPSS (v.24) software. Considering the few number of population samples, some parameters failed in the normality test, so all parameters were analyzed using non-parametric statistical tests. Wilcoxon Signed Rank Test and Friedman Test were the nonparametric alternatives used to compare means between study time points. Paired- samples T-Test was used as Post-hoc pairwise comparisons to determine the exact differences between the time pairs [30]. Spearman correlation was performed in order to find significant correlations between performance measures, psychological variables and physiological data (ECG-related measures); and statistical significant differences between pre and post-stress conditions, concerning those variables. Median reaction time (RT) values were considered instead of mean values, because RTs are not normally distributed with a longer tail of slow compared with fast responses [31].

#### 2.4.2 ECG Data

In order to extract heartbeat information from the ECG recordings, a software with an ECG analyzer was used (software from Biodevices S.A - the same commercialized by this company to cardiology specialists). This software has an algorithm based on the one developed by Pan Tompkins [32] incorporating ECG physiological filters to detect the “R” points of the ECG waveform. Using this analyzer, the RR interval (time between two consecutives “R” peaks in the ECG) was extracted.

A simple verification according to Clifford *et al.* [33], was made to confirm if all the RR intervals were physiologically valid. The RR intervals that have physiological acceptance are named normal-to-normal (NN) intervals.

Following the guidelines presented by the task force of the European Society of Cardiology and the North American Society of Pacing and Electrophysiology [34] different HRV time and spectral domains parameters were used (see Table **1**). Methods in the time domain define the intervals between successive normal QRS complexes (the Q-wave, R-wave, and S-wave). Measurements in the frequency domain provide information of how power (variance) distributes as a function of frequency. For time domain parameters, we used the Average of NN intervals (AVNN) and Standard Deviation of all NN (SDNN), expressed in milliseconds (ms) as well as the percentage of the number of pairs of successive NNs that differ by more than 20 or 50 ms compared to the total number of NN intervals, normally referenced to as pNN20 and pNN50, respectively [34]. The root mean square of differences between successive Rhythm-to-Rhythm (RR) intervals (RMSSD) was also used considering that it describes short-term HR variations. For extraction of spectral parameters the Lomb Periodogram was implemented [35]. For frequency domain analysis, we used both the Low Frequency (LF) component defined between 0.04-0.15 Hz as well as the high frequency (HF) component (0.15-0.4 Hz) and computed their ratio - LF/HF. A recent systematic review conducted by Castaldo et al. [12] in order to find the more reliable HRV features trends during stress (see Table **1**) suggested that four measures—AVNN, RMSSD, pNN50—in time and non-linear domains, resulted in being significantly depressed during stress. SDNN were also reduced in the majority of studies. Even though pNN20 is not frequently used in the literature, this metric was considered in our study, due to the interesting results obtained by Schaaff *et al.* [36]. The ratio between low and high frequency (LF/HF) showed to be significantly increased, suggesting a sympathetic activation and a parasympathetic withdrawal during acute stress.

#### 2.4.3 Cognitive Performance

Cognitive performance was analyzed considering the following Reaction Time (RT) related variables: median; minimum; maximum; standard deviation and the number of missed targets (missings) when performing CRTT. Performance results in terms of the number of correct responses versus total number of responses was not accessed for this experiment in specific, due to its low degree of complexity and habituation effects that could occur between the transition from the first time that participants performed the task (CRTT1) to CRTT2. Z-score values were considered regarding cognitive performance (RT-related variables) group statistical analysis.

## 3 RESULTS

### 3.1 Psychological Stress Scores

**Fig. (3):**
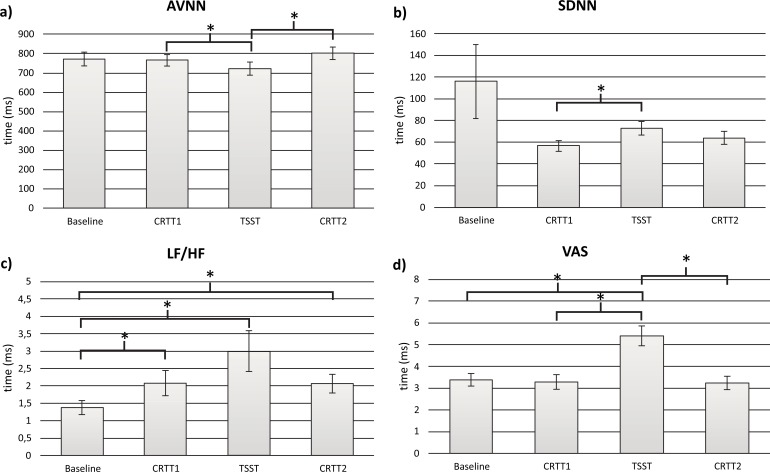
Mean statistical significant differences for ECG-derived measures – **a)** AVNN; **b)** SDNN; **c)** LF/HF and subjective stress measures - **d)** VAS across study time points (p<.005; Friedman test). *Represents statistical significant differences (p<.005; post-hoc pairwise comparisons).

To test the Hypothesis 1, stress self-perceptions were firstly analyzed to understand if the acute stress task was self-perceived as stressful. Friedman Test was used to analyze VAS scores along the protocol. Results indicated that there was a statistically significant change in perceived stress between the four time points of the study: baseline, CRTT1, TSST and CRTT2 - χ2 (2, n = 21) = 28.95, *p* < .001. Post-hoc pairwise comparisons showed that perceived stress significantly increased from baseline (M=3.39) to TSST (M=5.41) and from CRRT1 (M=3.29) to TSST (M=5.41). A significant decrease was found from TSST (M=5.41) to CRTT2 (M=3.24) (Fig. **3d**, Table **2**).

Wilcoxon Signed Rank Test was used to analyze STAI 6-item short-form data. Results revealed a statistically significant reduction of stress levels, from the beginning of the protocol to the end of the protocol, z = –2.24, *p* = < .05, with a medium effect size (r = .35). The median score on STAI 6-item short-form decreased from the beginning of the protocol (M = 2.02) to the end of the protocol (M = 1.76) (Table **2**).

### 3.2 ECG Data

To test the Hypothesis 1, regarding the physiological impact of acute stress, ECG data was obtained from 17 participants out of the 21. Four ECG recordings were removed due to problems in server data storage. Physiological baseline data collected at the 5 first minutes of the protocol were compared with the three important time points of the study: the pre-stress condition - CRTT1, the stress condition – TSST; and the post stress condition - CRTT2 using 5 minutes windows length. The parameters used for these analysis were time domain: AVNN; SDNN; RMSSD; pNN20; pNN50 and frequency –domain: LF/HF. Friedman Test was used to understand if there were statistically significant differences in these prameters across the four time points of the study (Fig. **3a**). Regarding AVNN, significant differences were found between the study time –points: χ2 (2, n=17) =16.55; p < .005. Post-hoc pairwise comparisons showed that AVNN significantly decreased from CRTT1 (M=765.65) to TSST (M=721.47) and then it increased from TSST (M=721.47) to CRTT2 (M=800.60). Inspection of the mean values showed that AVNN achieved the lowest value during TSST (M=765.65) (Fig. **3b**). Regarding the SDNN, χ2 (2, n=17) =15.00; p < .005, post-hoc pairwise comparisons showed that SDNN significantly increased from CRTT1 (M=56.722) to TSST (M=72.74). For LF/HF - χ2 (2, n=17) =19.94; p < .001, post- hoc pairwise comparisons showed that LF/HF significantly increased from baseline (M=1.38) to CRTT1(M=2.08), baseline (M=1.38) to TSST (M=3.00) and baseline (M=1.38) to CRTT2 (M=2.07). The highest value was obtained during TSST (M=3.00) (Fig. **3c**). In the time domain, with the exception of AVNN and SDNN, results from other parameters do not show any significant differences. However, there is a tendency for a lower RMSSD, pNN20 and pNN50 during the stress condition, when compared to the other moments.

### 3.3 Cognitive Performance

Regarding the subpopulation analyzed in terms of the influence of acute stress in cognitive performance when performing a simple Choice Reaction Time Task (CRTT), 19 participants were considered. Two participants were removed due to technical problems when saving data from the RT task.

To test Hyphotesis 2, some RT-related tasks and measures were statistically analysed. Despite cognitive performance in terms of RT-related measures and missed trials,apparently impaired by TSST; median RT, maximal RT, minimum RT and percentage of missing trials increased in post-stress condition -, none of these variables were shown to be significantly different between pre- and post-stress conditions (Table **3**).

**Fig. (4):**
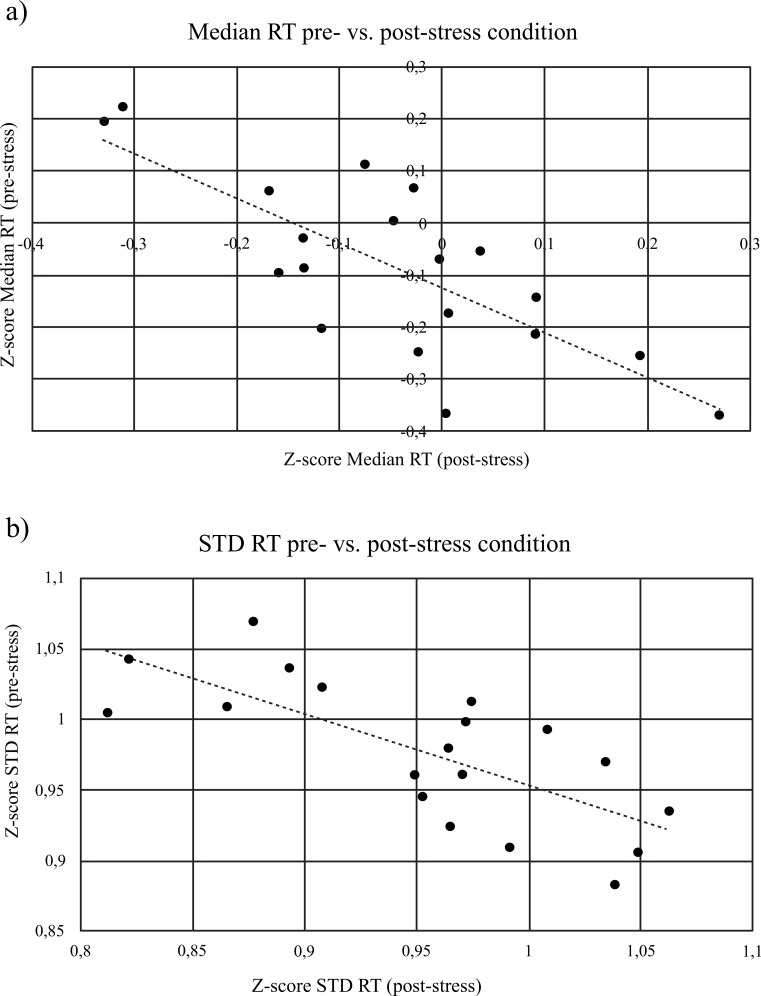
RT-related measures evolution between pre- and post-stress conditions. **(a)** Graphic representing the relation between z-score Median RT pre- (CRTT1) and post-stress (CRTT2) conditions and corresponding fit line. **(b)** Z-score standard deviation values (SD) of RT relation between pre- and post-stress stages and fit line.

Significant correlations were found between some of the cognitive performance variables using Spearman´s rank order correlation (Table **4**) as explained below. A strong and negative correlation was found for median RT values between the CRTTs performed before and after stress condition, r = -.71, *p* < .001 suggesting that, the faster the subjects responded to targets in the CRTT1 (pre-stress condition), the slower they were in CRTT2, which is reinforced by the fact that Median RT median values across participants were higher after TSST compared to the previous finding (Fig. **4a**). A strong and positive correlation was also found between Max RT values and percentage of Missings only in the CRTT2, r =.53, *p* < .01 (Table **4**). Standard deviation of RT values showed a decrease in CRTT2 while stress levels increased, even though this change was not statistically significant. However, three significant correlations were found regarding this variable.

Firstly, there was a negative and strong correlation between Min RT and standard deviation RT values only in CRTT2, r = -.67, *p* < .01, which increased and decreased respectively, after the stress condition. Secondly, standard deviation RT variable was shown to be negatively and strongly correlated between pre- and post-stress condition, r = -.70, *p* < .01 (Table **4**). Thirdly, the difference of Min RT values between CRTT2 and CRTT1 was negatively and strongly correlated with the difference of RT standard deviation values between CRTT2 and CRTT1, r = -.63, *p* < .01 (Table **4**) and considering that Min RT values increased after TSST and RT standard deviation values decreased after TSST, the proposed protocol showed that acute stress events are able to hamper response speed, despite turning response time frequency range narrower (Fig. **4b**).

## 3 DISCUSSION

This study investigated the effects of stress on cognitive performance of firefighters and whether this effect was related to the autonomic response to stress, using a biomonitoring platform. Several HRV parameters were analysed in order to identify those that change significantly under the influence of stress.

Our first hypothesis indicated that stress would have an impact on participants physiology, particularly a reduction in some ECG time-domains—AVNN, SDNN, RMSSD, pNN20 and pNN50 and an increase in ECG frequency-domains— particularly LF/HF. In order to test the abovementioned hyphothesis, we analyzed psychological self-reported data in order to confirm if the chosen stress paradigm was in fact stressful for the participants. Hence, the TSST was chosen, based on previous research that indicated this task as a gold stress standard task [29, 37]. Our results based on stress VAS results, along with the some HRV parameters showed that the selected stress test indeed evoked stress and was thus appropriate for the aim of this study. Accordingly, the present study shows that short-term psychological stress caused significant changes in some time domain measures – AVNN and SDNN and frequency domain parameters – LH/HF between time points of the study. The decrease in AVNN from CRTT1 (pre-stress) to the TSST (stress condition), suggested that the stimulus caused a higher activation of the cardiovascular system, preparing the body to rapidly react (fight-or-flight response) [38]. Hence, lower values are associated with stress responses [12]. Regarding SDNN, there was a significant increase from CRTT1 to TSST. This is an unexpected result, because according to the literature in the area, acute stress tends to cause a decrease in SDNN [12]. These findings are similar to those by, Schubert *et al*. [39] in a study examining the stress effects on HRV, in 50 healthy subjects using a short-term stressor reactivity assessed with a speech task. Among other findings, results suggested that SDNN increased during the stress condition. The authors proposed that talking loud and particularly, doing stressful mental arithmetic’s were associated with a slowing in respiratory rate and a relative reduction in ventilation, and increase in SDNN. Such evidence on the influence of speech-related respiratory patterns on linear HRV measures could explain why in the current study the stress condition (TSST- that also includes a speech task and an arithmetic task) was associated with decreases in SDNN, independently of changes in parasympathetic modulation of HR.

The remaining time domain features (RMSSD, pNN20 and pNN50) showed a consistent but non-significant decrease during the stress condition, which is also compatible with a stress response [40, 41]. This might be explained by the limited number of subjects. Within the frequency domain, a significant increase in the LF/HF ratio during the stress condition was also found, indicating the marked effect of stress on sympathovagal balance [42]. Higher values reflect the domination of the sympathetic system over the parasympathetic one, typical of stress responses [43]. A recent study conducted with air traffic controllers using a similar method also found statistically significant differences in some HRV metrics (AVNN, SDNN and LF/HF), reinforcing therefore the discriminatory power of these metrics for the stress quantification using HRV measurements [44]. These physiological results, gathered by the wearable ECG device, were concordant with an elevation of self-perceptions stress (VAS) in the stress condition, which returned to baseline at the end of the protocol, confirming therefore our hyphothese 1, with the exception of SDNN that increased with stress.

Our second hypothesis porposed that acute stress would impair cognitive performance, by increasing the RTs and the missing answers. Our results confirm this hyphotheses, showing that performance was impaired after stress. Accordingly, averaged RT median values across participants were negatively correlated between pre-and post-stress condition, showing that acute stress served as a turning point for the firefighter’s cognitive performance along the protocol. Additionally, significant correlations were found between some of the cognitive performance variables, indicating that TSST has negatively influenced the ability to respond quickly and effectively to targets. Particularly, performance was impaired precisely in terms of maximal RT and Missings percentage. Additionally, the fact that those two variables were positively correlated in CRTT2, reinforces the decline in performance induced by the increased stress levels after TSST. Considering that RT variability values also decreased after stress, it has been suggested that probably, the activation of sympathetic nervous system in acute stress conditions leads to a less variable RT response as already stated by previous studies not directly compatible with an improvement response judgment ability [45]. In fact, perception ability was effectively impaired by stress levels in this case, leading to more missed trials by participants in CRTT2. It can be concluded that stress negatively influenced the cognitive performance of participants. Accordingly, a study conducted with 21 firefighters in order to understand stress during real emergency scenarios and its impact on cognitive performance found that cognitive performance was impaired immediately after their training period and after a short delay. In line with these findings, it was also found in the current study that despite the fact that after the stress condition, and during the CRTT2, HRV parameters showed a trend to “stabilize”, participants presented an impairment on their cognitive performance, by decreasing their attention levels. Similarly, in a study conducted with 23 healthy participants aiming to investigate the effects of TSST on impaired attention and working memory, it concluded that abnormalities in attention persisted for at least 30 minutes following the stressor [37].

These results enhance even more the negative impact of stress levels on cognitive abilities in short and long terms, particularly for firefighters, who are constantly suffering from stressful/high strain events.

This study is not without limitations. Particularly, the reduced sample size, the trasnversal evaluation, the absence of a control group and the focus on only one simple cognitive task. Additionally, despite this task is a gold standard stress task in the literature, it does not represent the real world stressors faced daily by firefighters. Despite these, the current study is unique in terms of the specific population under study and the methodology used, providing an important insight into the direct effects of stress and allowing future avenues for new perspectives on the treatment and prevention of stress-related diseases, including a more personalized health and wellbeing approach.

Additionaly, the use of a medical certified wearable and non-invasive device, that collects clinical-grade ECG is also an asset in this study. Our scope is to deploy, in the future, this wearable platform during real situations, using the same population. The current results will serve as a stress groundtruth, where machine learning techniques will be implemented for the development of algorithms that will be used in early stress detection.

## CONCLUSION

Findings from the current study suggested a relationship between stress and cognitive performance. Particularly, after an increase in the stress levels, the cognitive performance of firefighters decreased. These results support the need to further understand firefighters stress and cognitive functioning in order to prevent wrong decisions under pressure. Applied interventions should mitigate vulnerability to stress, by preparing and training firefighters. Moreover, prevention programs (*e.g*., mindfulness training) should also be developed and adapted to this population needs.

Regarding the autonomic response to stress we have identified a significant change in some HRV parameters under the influence of stress. Particularly, AVNN and LF/HF ratio seems to be very promising features for short-term stress classification based on HRV as classification results. This information could be of great help for researchers in the development of qOHealth technology, like the design of on-line platforms and devices that will help integrate psychophysiological stress information. Consequently, fire administrators and the firefighters themselves will benefit from the use of real time information. Acknowledging that critical decisions need to be made quickly on the spot, it seems advantageous to have complementary data to support decisions. As an example, awareness of firefighter’s real stress state in a particular situation, might be useful to make informed decisions on resting periods, task schedules or even on self-regulation strategies. This might be suitable information to consider for the professional task assignment (e.g., providing more resting periods). Additionally, future studies should consider the use of this type of bio monitoring platforms of stress during real events.

## Figures and Tables

**Fig. (1):**
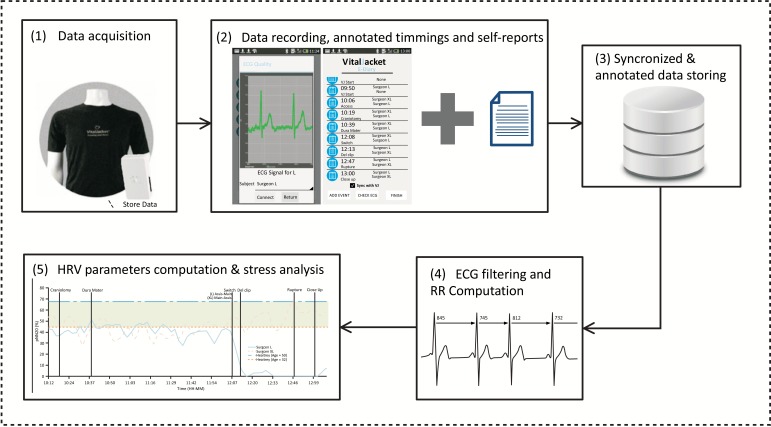
System block diagram explaining the bio monitoring platform architecture.

**Fig. (2):**
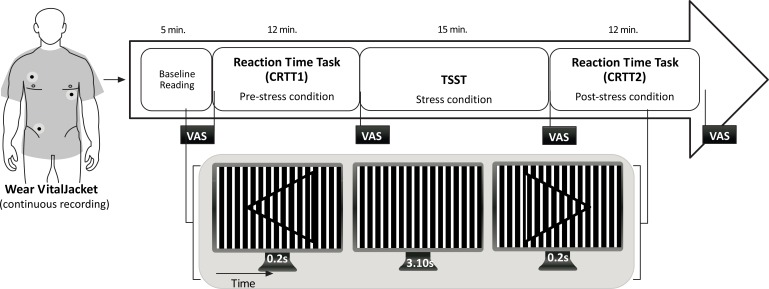
Diagram of the protocol. VAS - Visual Analogue Scales. TSST - Trier Social Stress Test.

**Table 1:** Descriptions of HRV measures and their tendency under stress [12].

Domain	Measure	Description	Features trend under stress
Time-domain	AVNN	Average of NN intervals (ms)	↓
-	SDNN	Standard Deviation of all NN intervals (ms)	↓↑
-	RMSSD	Root mean square of differences of successive NN intervals (ms)	↓
-	pNN20	NN variations above 20 ms (%)	↓
-	pNN50	NN variations above 50 ms (%)	↓
Frequency-domain	LF/HF	Describes the ratio of Low Frequency and High Frequency power bands.	↑

**Table 2:** Mean and standard deviation of self-reported measures of stress.

Psychological instruments	Mean ± SD
STAI 6-item beginning of the protocol	2.02 ± 0.47
STAI 6-item end of the protocol VAS baseline VAS CRTT1 VAS TSST VAS CRTT2	1.76 ± 0.39 3.39 ± 1.16 3.29 ± 1.36 5.41 ± 1.87 3.24 ± 1.25

**Table 3:** Cognitive performance results regarding CRTT performed before and after the stress condition in terms of Reaction Time (RT) and percentage of missed trials (Missings).

PARAMETER	CRTT1	CRTT2
Median RT (s)	0.482 ± 0.042	0.486 ± 0.044
Max RT (s)	0.693 ± 0.073	0.698 ± 0.095
Min RT (s)	0.323 ± 0.039	0.336 ± 0.035
SD RT (s)	0.072 ± 0.012	0.071 ± 0.015
Missings (%)	3.637 ± 3.514	4.197 ± 4.266

Max – Maximal; Min – Minimal; SD – Standard Deviation

**Table 4:** Significant correlations values between the cognitive performance variables (median RT, Maximal RT, Min RT and Standard deviation of RTs) obtained during the two reaction time tasks (CRTT1 and CRTT2).

**Correlated variables**	**Spearman r value**
Median RT CRTT1 *vs.* Median RT CRTT2	-.71***
Max RT CRTT2 *vs.* Missings CRTT2	.53*
Min RT CRTT2 *vs.* SD RT CRTT2	-.67**
SD RT CRTT1 *vs.* SD RT CRTT2	-.70**
Diff Min RT *vs.* Diff SD RT	-.63**

Diff Min RT = Min RT CRTT2 – Min RT CRTT1; Diff SD RT = SD CRTT1 – SD CRTT2; *p < .05; ** p < .01; *** p < .001

## LIST OF ABBREVIATIONS

ANS: = Autonomic Nervous System
AVNN: = Average of NN intervals
CRTT: = Choice Reaction Time Task
ECG: = Electrocardiogram
HF: = High Frequency
HR: = Heart Rate
HRV: = Heart Rate Variability
IBM SPSS: = Statistical Package for Social Sciences software
LF: = Low Frequency
LF/HF: = Ratio of Low Frequency and High Frequency power bands
NN intervals: = Normal-to-Normal intervals
pNN20: = NN variations above 20 ms
pNN50: = NN variations above 50 ms
qOHealth: = Quantified Occupational Health
RMSSD: = Root Mean Square of differences of successive NN intervals
RT: = Reaction Times
RR intervals: = Rhythm-to-Rhythm intervals
SDNN: = Standard Deviation of all NN intervals
STAI: = Spielberg State-Trait Anxiety Inventory
TSST: = Trier Social Stress Test
VAS: = Visual Analogue Scales
